# Investigating the Role of the microRNA-34/449 Family in Male Infertility: A Critical Analysis and Review of the Literature

**DOI:** 10.3389/fendo.2021.709943

**Published:** 2021-07-01

**Authors:** Konstantinos Pantos, Sokratis Grigoriadis, Penelope Tomara, Ioanna Louka, Evangelos Maziotis, Agni Pantou, Nikolaos Nitsos, Terpsithea Vaxevanoglou, Georgia Kokkali, Ashok Agarwal, Konstantinos Sfakianoudis, Mara Simopoulou

**Affiliations:** ^1^ Centre for Human Reproduction, Genesis Athens Clinic, Athens, Greece; ^2^ Laboratory of Physiology, Medical School, National and Kapodistrian University of Athens, Athens, Greece; ^3^ Assisted Reproduction Unit, Second Department of Obstetrics and Gynecology, Aretaieion Hospital, Medical School, National and Kapodistrian University of Athens, Athens, Greece; ^4^ American Center for Reproductive Medicine, Cleveland Clinic, Cleveland, OH, United States

**Keywords:** male infertility, idiopathic male infertility, micro-ribonucleic acids (microRNAs), miR-34/449, spermatogenesis, ciliogenesis, biomarkers, personalized medicine

## Abstract

There is a great body of evidence suggesting that in both humans and animal models the microRNA-34/449 (miR-34/449) family plays a crucial role for normal testicular functionality as well as for successful spermatogenesis, regulating spermatozoa maturation and functionality. This review and critical analysis aims to summarize the potential mechanisms *via* which miR-34/449 dysregulation could lead to male infertility. Existing data indicate that miR-34/449 family members regulate ciliogenesis in the efferent ductules epithelium. Upon miR-34/449 dysregulation, ciliogenesis in the efferent ductules is significantly impaired, leading to sperm aggregation and agglutination as well as to defective reabsorption of the seminiferous tubular fluids. These events in turn cause obstruction of the efferent ductules and thus accumulation of the tubular fluids resulting to high hydrostatic pressure into the testis. High hydrostatic pressure progressively leads to testicular dysfunction as well as to spermatogenic failure and finally to male infertility, which could range from severe oligoasthenozoospermia to azoospermia. In addition, miR-34/449 family members act as significant regulators of spermatogenesis with an essential role in controlling expression patterns of several spermatogenesis-related proteins. It is demonstrated that these microRNAs are meiotic specific microRNAs as their expression is relatively higher at the initiation of meiotic divisions during spermatogenesis. Moreover, data indicate that these molecules are essential for proper formation as well as for proper function of spermatozoa per se. MicroRNA-34/449 family seems to exert significant anti-oxidant and anti-apoptotic properties and thus contribute to testicular homeostatic regulation. Considering the clinical significance of these microRNAs, data indicate that the altered expression of the miR-34/449 family members is strongly associated with several aspects of male infertility. Most importantly, miR-34/449 levels in spermatozoa, in testicular tissues as well as in seminal plasma seem to be directly associated with severity of male infertility, indicating that these microRNAs could serve as potential sensitive biomarkers for an accurate individualized differential diagnosis, as well as for the assessment of the severity of male factor infertility. In conclusion, dysregulation of miR-34/449 family detrimentally affects male reproductive potential, impairing both testicular functionality as well as spermatogenesis. Future studies are needed to verify these conclusions.

## Introduction

According to the World Health Organization (WHO) infertility is defined as the inability to achieve a pregnancy following at least twelve months of unprotected sexual intercourse (1, 2). Recent data demonstrate that the prevalence of infertility is very high, affecting 8-12% of couples of reproductive age worldwide (1–3). Infertility could be attributed to both female and male factors, detrimentally affecting reproductive potential. Male factor infertility represents the sole infertility aetiology for 20-30% of infertile couples. Additionally, and in combination with female etiology, male infertility presents as a contributory factor for 50% of infertility cases (3, 4). Male infertility is a multifactorial condition caused by a wide variety of congenital, acquired, and idiopathic factors (2, 3, 5). Moreover, several environmental stressors and life-style parameters, including nutrition, alcohol consumption and smoking, could detrimentally affect male reproductive system functionality and dynamic (6). However, and despite advances in the field of human reproduction, the exact infertility aetiology remains unknown regarding 30% of infertile men, highlighting the need for a more accurate and a more precise understanding of the molecular mechanisms involved in the pathogenesis of male infertility (7).

Aiming to instigate, present, discuss and critically analyze novel possible molecular targets of male infertility, authors of the present review focused their attention on the role of micro-ribonucleic acids (microRNAs). Micro-ribonucleic acids are a class of small non-coding ribonucleic acids (RNAs), constructed by an average sequence of 22 ribonucleotides (8). Their loci is commonly found in transcription units and are produced following the transcription of these gene regions. Moreover, microRNAs are commonly located in clusters throughout the genome, except for the Y chromosome (9, 10). Generally, microRNAs act as epigenetic modifiers regulating gene expression in the post-translation level, causing translation repression and/or degradation of their mRNA targets (11). Thus, microRNAs are described as master regulators of several biological processes both in the cellular and the organism level (8). Micro-ribonucleic acids are involved in cell cycle regulation, in cell programming as well as in cell differentiation and tissue formation (12). Moreover, microRNAs exert a pivotal role on embryo development, on organ formation as well as on growth (13–15). Due to their pivotal role on regulation of cellular processes, microRNA dysregulation is considered to be directly associated with the pathogenesis of several diseases and pathophysiological conditions, including developmental diseases, cancer and infertility (16).

This narrative review is focused on identifying and underlying the possible role of a specific microRNA family, namely microRNA‐34/449 (miR-34/449) on male infertility pathophysiology. Briefly, the miR-34/449 family is comprised by six highly conserved microRNAs, namely miR-34a/b/c and miR-449a/b/c, respectively (17). The miR-34a gene is located on chromosome 1p36.22 (18). The other five family members are classified into two functional clusters, namely miR-34b/c cluster and miR-449a/b/c cluster, respectively (17). Members of both of these clusters are co-expressed in the testis and present with common characteristics (19). The miR-34b/c cluster is located on chromosome 11q23.1 and the miR-449a/b/c cluster is originated from polycistronic transcripts encoded on chromosome 5q11.2 and more specifically in the second intron of the cell division cycle 20b (cdc20b) gene (20). Despite the fact that the members of the miR-34/449 family are transcribed from different gene loci, these present with similar mature sequences and share an identical ‘‘seed sequence’’ (21, 22). As a result, these microRNAs are sharing common features and properties. In both animals and humans, the members of miR-34/449 family exert several significant functions, including cell cycle regulation as well as regulation of cell differentiation and functionality. These microRNAs are expressed in several tissues and organs such as in airway epithelium, in ovaries, in fallopian tubes, in epithelial cells located on the surface of the ventricular system of the brain and finally and most importantly in testis (19, 23–25). All these tissues are sharing a common dominator: among their different cell types, these tissues contain ciliated cells and/or flagellate cells, such as spermatozoa. Considering the topology of their expression, it becomes clear that miR-34/449 members constitute master regulators of the ciliated cells’ differentiation and function (22, 26). Most importantly, considering the topic of the present review, there is increasing evidence suggesting that miR-34/449 family members play crucial roles in testicular functionality as well as in regulating spermatogenesis and mature spermatozoa formation (19, 25, 27). The essential role that miR-34/449 family members appears to play in the male reproductive system served as the incentive for authors to proceed to this critical analysis, while the rational for this study was fueled by the need to provide further in depth analysis on male infertility.

In summary, data presented from several studies both in humans and animal models indicate that miR-34/449 family members play crucial roles during spermatogenesis by regulating spermatozoa maturation and testicular functionality. It is suggested that miR-34/449 dysregulation is related with several aspects of idiopathic male infertility ranging from oligozoospermia to non-obstructive azoospermia (NOA). This critical analysis aims to highlight the potential mechanisms *via* which miR-34/449 dysregulation could lead to male infertility, highlighting the role of these molecules as potential biomarkers and possible therapeutic targets for improving management of male infertility in the era of personalized and precision medicine.

## Multiciliated Cells and Normal Testicular Functionality

Prior to elaborating on the role of miR-34/449 family members on male infertility it is significant to understand their contribution in proper development and function of testicular multiciliated cells. Cilia are dynamic microtubule-based cell organelles that extend outside the cell body (28). These organelles are highly conserved among eukaryotes and consist of the basal body, which resides in the apical surface inside the cell and the extracellular axoneme structure (29). Cilia can be motile or immotile exerting different cell functions. Generally, almost all human cells possess a single-non motile cilium, commonly characterized as primary cilium, which plays crucial roles mainly with regard to cell signaling serving like a sensor (30, 31). However, there is a class of hostile specialized differentiated epithelial cells, which possess multiple motile cilia. These cells, which project multiple cilia on their apical surface, are commonly referred to as multiciliated cells (32–36). The multiple cilia of these cells are generally moving in a synchronous wave-like manner to facilitate movement of luminal contents in several tissues and organs, including the airway epithelium, the ventricular system of the brain as well as the spinal column (19, 23–25, 37). Interestingly, multiciliated cells are observed in both the female and male reproductive system (38).

Considering the female reproductive system, multiciliated cells are mainly located in the epithelium of the fallopian tubes and *via* the synchronous wave-like motion of their multiple cilia these oviduct epithelial cells assist the oocyte and embryo passage towards the uterus (39). With regards to the male reproductive system, data indicate that multiciliated cells are present in the epithelium of the efferent ductules (27, 40). Similarly to the fallopian tubes, efferent ductules are small tubes *via* which the immature immotile spermatozoa are transported from the rete testes to the caput epididymis, where the final maturation of the spermatozoa is taking place (27, 40). In the efferent ductules immature spermatozoa are suspended in an abundance of seminiferous tubular fluid, the great amount of which is reabsorbed prior to reaching the caput epididymis (41). Seminiferous tubular fluid reabsorption is of paramount importance since immature spermatozoa should reach the caput epididymis in high concentrations in order for the final spermatozoa maturation to be achieved (27). The multiciliated cells of the efferent ductules seem to play crucial roles in both immature spermatozoa transportation into the epididymis, as well as in the successful reabsorption of the seminiferous tubular fluids (27). Recently, published data demonstrate that cilia in the efferent ductules are not moving in a synchronous wave-like manner, but in contrast are performing whip-like beatings and continually change the direction of the fluids’ flow, generating turbulence, which is turn keeps spermatozoa suspended in the seminiferous tubular fluids (27, 42). Therefore, it becomes evident that the multiciliated cells located in the efferent ductules are significant contributors for normal testicular functionality.

## MiR-34/449 Family: A Requirement for Multiciliated Cell Differentiation

Despite the fact that the multiciliated cells are present in several different organs and tissues, data demonstrate that cilia formation, being a differentiation process called ciliogenesis, is taking place *via* shared molecular mechanisms. During the first stage of ciliogenesis, progenitor cells should exit the cell cycle to acquire the multiciliated phenotype. Several endocrine, paracrine, mechanical and chemical stimuli could induce ciliogenesis (43). In progenitor undifferentiated basal cells ciliogenesis is initiated by the amplification of centrioles, representing the second stage of the whole process. This event is regulated by two independent pathways, the canonical parental centriole dependent pathway, and the deuterostome dependent pathway (44). Centriole abundance is proportional to the surface area and is not associated with cell volume (45). Following the amplification stage, centrioles move to the apical membrane of the cell, where they dock and maturation is achieved giving rise to basal bodies (28). The axoneme is the scaffold of the cilia and is a dynamic structure, which originates from the basal bodies. The axoneme is comprised of several proteins, including A and B tubulins and dynein (46).

Considering the role of miR-34/449 family in ciliogenesis, there is a great amount of evidence suggesting that these microRNAs share common developmental roles, serving as master regulators of the ciliogenesis process in several tissues and organs, including the testis (19, 23–25). This becomes evident considering that the miR-34/449 family members are overexpressed during ciliogenesis in vertebrates and constitutes the most highly expressed microRNA family in the ciliated epithelia (20). Moreover, data indicate that genetic ablation of both miR-34b/c and miR-449a/b/c clusters could detrimentally affect ciliogenesis *via* dysregulation of several genes involved in cell cycle regulation. In the absence of miR-34/449 several genes encoding cell cycle regulatory proteins, namely cyclin-dependent kinases (CDKs), are upregulated resulting to impaired ciliogenesis. These proteins keep progenitor cells to a proliferative stage, rendering them incapable of exiting the cell cycle in order to adopt the multiciliated phenotype (12). It is well established that the members of the miR-34/449 family present with pro-apoptotic and anti-proliferative properties, which are exerted by their ability to silence the expression of several proteins promoting cell proliferation, including CDKs as well as cyclins (47–49). It seems that during ciliogenesis, these properties are also important for the successful differentiation of the multicillia epithelium in several tissues, including the testis.

In order to better understand the significant role of the miR-34/449 family during ciliogenesis it is of high significance to present the molecular pathways mediating respective actions. Data originating from both animal models and humans indicate that the members of the miR-34/449 family work synergistically during ciliogenesis. Firstly, during early ciliogenesis these microRNAs suppress both Notch and bone morphogenetic protein (BMP) signaling pathways, enabling progenitor cells to exit the cell cycle. Moreover, Notch and BMP suppression mediates centriole amplification and migration of the amplified centrioles to the apical surface of the progenitor cells (20, 50, 51). Several studies indicate that Notch suppression stands as a prerequisite condition in order for the progenitor cells to be successfully differentiated into multiciliated cells. Experiments performed, employing both Xenopus epidermis and cultured human aortic endothelial cells (HAEC), demonstrate that miR-449a/b/c cluster’s members can directly suppress the Nocth1 receptor as well as its ligand delta like ligand 1 (DLL1), promoting ciliogenesis. In contrast, when Notch1 and DLL1 messenger RNAs (mRNAs) were protected against the miR-449a/b/c, an impaired ciliogenesis was observed (20). These data indicate that miR-34/449 family is required for successful determination of the multiciliated cell fate by silencing significant pathways, including Notch and BMP signaling pathways.

Members of the miR-34/449 family are not only required at the early stages of the ciliogenesis, but also act as significant regulators of the following steps. There is evidence suggesting that miR-34/449 family members work together for the formation of the apical actin meshwork as well as for the formation and the maturation of the basal bodies. Data indicate that miR-34/449 family members can directly or indirectly suppress the mRNA of the RAS related protein (R-Ras), controlling the redistribution of Filamin A protein on the apical actin meshwork of the multiciliated cells. It seems that these specific microRNAs serve as regulators of the apical actin meshwork’s architecture, which is a crucial structure necessary for basal body anchoring (50, 51). In addition, miR-34/449 members also control cilia biogenesis *via* the regulation of the cp110 mRNA. The cp110 protein is a regulatory molecule necessary for both cilia formation and function. During ciliogenesis, cp110 levels should be accurately controlled to enable proper formation and function of apical actin meshwork as well as of basal bodies. Strong correlation of the cp110 protein with the miR-34/449 family members is highlighted considering that both cp110 and miR-34/449 can be regulated by the same transcription factors. However, the miR-34/449 members can post-transcriptionally control expression of the cp110 protein, and thus they can regulate optimal cp110 proteins’ levels for the apical actin meshwork formation as well as for the basal bodies anchoring (22, 26, 52). Moreover, miR-34b can regulate levels of c-Myb protein. The c-Myb protein is a well-known transcription factor, which controls several genes involved in cell differentiation and cell survival (53–55). The c-Myb is also one of the master regulators of ciliogenesis, controlling expression of several ciliary-related genes, including Polo like kinase-4 (PLK4) and Stil (Scl/Tal1 interrupting locus) genes. Experiments performed in zebrafish kidney’s multiciliated cells indicate that the miR-34b/c-Myb signaling serves as an important regulator of centriole migration and docking. Genetic ablation of miR-34 or c-Myb overexpression can equally impair the normal centriole migration and docking. In the middle kidney ducts of c-Myb mutants, a fewer number of centrioles lining the apical membrane of the multiciliated cells was observed, indicating that optimal levels of the c-Myb transcription factor are required for proper centriole migration. Although the c-Myb transcript presents binding sites for the miR-34b, its regulation seems to be indirect and more date is required to elucidate whether miR-34b controls c-Myb levels (56). These data demonstrate that miR-34/449 members represent essential elements of the molecular mechanisms regulating apical actin meshwork formation as well as basal body anchoring.

## Impaired Testicular Multiciliogenesis and miR-34/449 Dysregulation

Several studies have been so far conducted in order to elucidate whether miR-34/449 dysregulation could impair ciliogenesis. Experiments performed in Xenopus epidermis as well as in ex vivo cultured cells demonstrate that the miR-449 cluster inhibition could impair multiciliated cell functionality by reducing the number of basal bodies (20). In contrast to the aforementioned observation, no significant change in the number of basal bodies was reported in the multiciliated cells of the respiratory tract of miR-34/449 knock out mice. However, defects in basal body migration and docking were observed as the majority of them were localized into the cytoplasm and did not migrate to the apical surface of the cells (22). Triple knock out (TKO) mice regarding all the miR-34/449 family genome loci presented with respiratory deficiency as well as with respiratory infections and were characterized by a phenotype resembling a condition called Primary Cilia Disorder (PCD). Further analysis revealed a dysfunction in generation of the multiciliated cells of the respiratory tracks, which was caused by defective basal body formation and docking (39). Same outcomes were observed regarding the ciliated epithelium of the reproductive tracks regarding both female and male mice, where ciliogenesis was also significantly impaired, leading to infertility (39). Similar findings were also reported by Otto et al., in mice in which both miR-34b/c and miR-449a/b/c clusters were genetically ablated. Impaired multiciliogenesis was observed in the respiratory and reproductive epithelia of both sexes. Histological examination of both embryos and adult mice confirmed that in the respiratory system, in the fallopian tubes as well as in the efferent ductules, ciliogenesis was defective. A microarray analysis of these tissues revealed that several cell cycle genes were significantly upregulated in both mRNA and protein levels, indicating the incapability of the progenitor cells to obtain the multiciliated phenotype. These finding were confirmed following proliferation assays, indicating that the miR-34b/c and miR-449a/b/c deficient mice displayed a higher fraction of proliferating cells compared to the wild-type controls (12). These data suggest that miR-34/449 family members are required for proper ciliogenesis, especially regarding respiratory and reproductive tracks epithelia. Their role is of paramount importance not only for the progenitor cells to acquire the ciliated phenotype, but also for the maturation, as well as for ensuring proper functionality of the differentiated multiciliated cells. Thus, alterations in their expression leads to both respiratory defects and infertility in both sexes.

In order to elaborate on the role of miR-34/449 family members on the female reproductive system’s physiology, it is of significance to note that genetic ablation of these microRNAs leads to impaired ciliogenesis in the epithelium of the fallopian tubes and subsequently to female infertility. These observations were confirmed following histological examination performed on TKO female mice which were treated with gonadotrophins. In these female mice, a significant reduction regarding the number of oocytes identified in the fallopian tubes was observed, a phenomenon which is likely attributed to the impaired multiciliogenesis of the fallopian epithelium (12, 22, 57, 58).

Regarding the male reproductive system, several studies demonstrated that genetic ablation of both miR-34b/c and miR-449a/b/c clusters results in spermatogenic impairment and subsequently to male infertility (27, 59, 60). Data originating from these studies indicate that male infertility is probably attributed to the impaired ciliogenesis of the efferent ductules. As previously highlighted, the motile cilia of the internal lumen of the efferent ductules present with unique properties and are required for the proper functionality of the testis (59). In 2019, Yuan et al. published high-quality evidence highlighting the significance of these unique properties of the multiciliated cells located in the efferent ductules. In this study, authors generated spermatogenic cell-specific and multiciliated cell-specific double miR-34 and miR-449 cluster knockout mice. Data demonstrated that in the absence of both miR-34 and miR-449 clusters, spermatogenic cells remained unaffected and these mice were totally fertile. In contrast, the mice of the multiciliated cell-specific double miR-34 and miR-449 cluster were infertile and presented with testicular atrophy. In addition, these mice exhibited impaired ciliogenesis of the efferent ducts due to the dysregulation of several genes contributing to ciliogenesis, namely Ccdc113 and Dnah6. In concordance with previous data, image analysis *via* electron microscopy revealed that the epithelium of the efferent ductules had fewer number of cilia, presenting with less motility compared with the wild-type mice (27). More specifically, data originating from this study indicate that genetic ablation of both the miR-34 and miR-449 clusters led to abnormal multicillia cell differentiation in the efferent ductules, which in turn led to sperm aggregation and agglutination, testicular tubular obstruction, impaired spermatogenesis, testicular atrophy and subsequently to male infertility (27).

In conclusion, altered miR-34/449 expression motifs can cause defective ciliogenesis in the efferent ductules epithelium, leading to progressive spermatogenic failure. In this case, spermatogenic failure is attributed to testicular dysfunction, mainly originating from impaired reabsorption of the seminiferous tubular fluids, which in turn leads to high hydrostatic pressure into the testis. High hydrostatic pressure progressively leads to testicular atrophy and subsequently to male factor infertility, which could range from OAT to azoospermia. These data demonstrate that clinicians should consider multicillia cell dysfunction and efferent ductules obstruction in cases where abnormal semen parameters and/or azoospermia co-exist with sperm aggregation and agglutination as well as with luminal enlargement (27).

## The Role of miR-34/449 Family in Spermatogenesis

**Figure 1:**
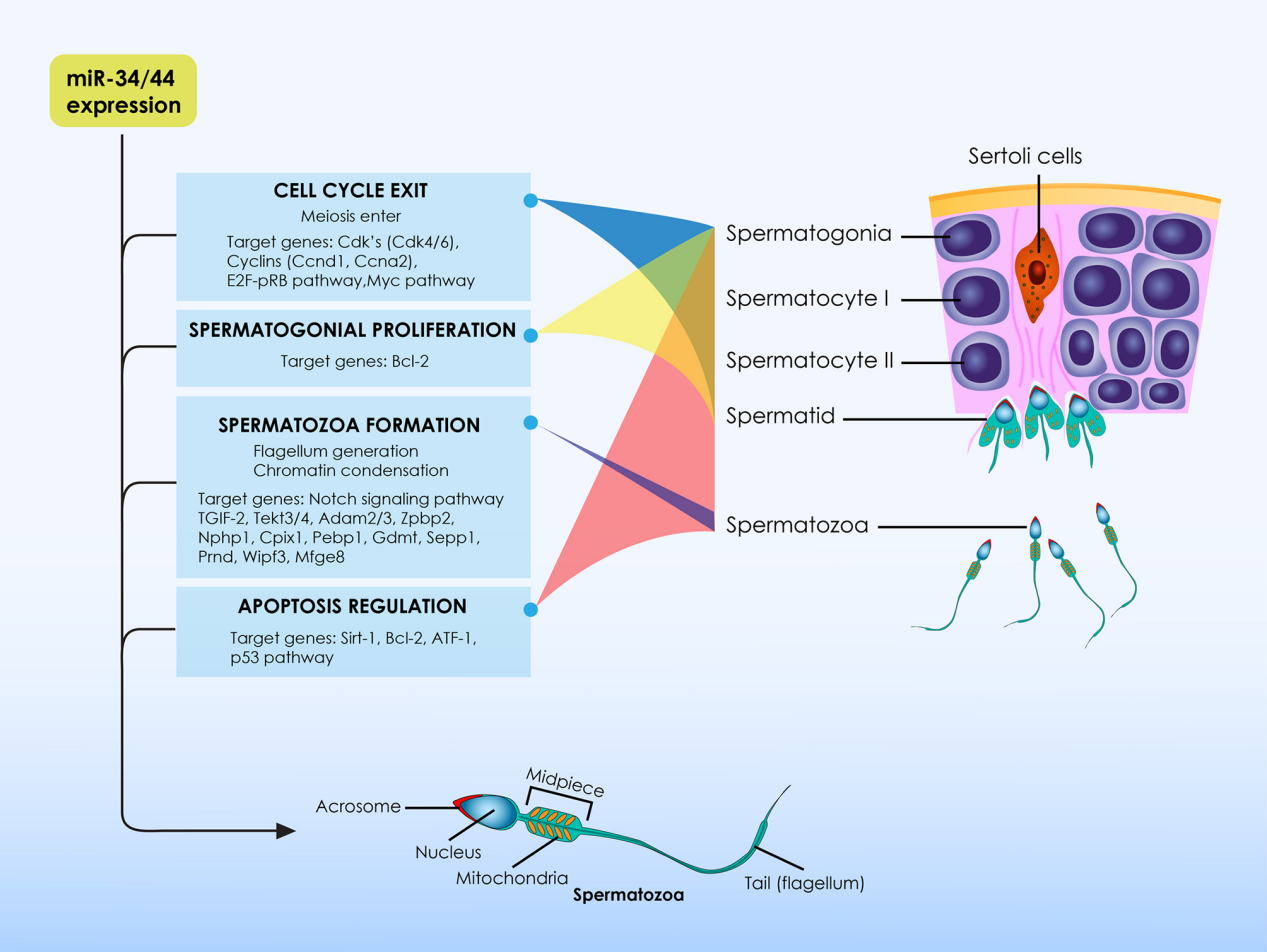
The role of miR-34/449 family members on the physiological events entailed during spermatogenesis. At the initial stages of spermatogenesis miR-34/449 family members target several transcripts of cell-cycle regulatory proteins, leading germ cells to exit the cell cycle in order for spermatogenesis to be initiated. They are considered to be meiotic specific microRNAs as their expression is relatively higher at the initiation of meiotic divisions. During spermatogenesis these microRNAs are essential molecules promoting normal spermatozoa formation *via the* differential regulation of expression levels of several spermatogenesis-related proteins depending on spermatogenesis stages. In addition, miR-34/449 family members do not only exert significant actions for the regulation of spermatogenesis, but also these molecules are essential for proper formation as well as for proper function of spermatozoa per se. Except from spermatogenesis, evidence also suggest that members of the miR-34/449 family exert significant anti-oxidant and anti-apoptotic properties and thus stand as significant elements of the testicular homeostatic mechanisms.

Taking into account the spermatozoa architecture, it may be hypothesized that the spermatozoon may in fact represent a highly differentiated type of ciliated cells. It is this exact hypothesis that drove the authors’ extrapolation that miR-34/449, ciliogenesis and spermatozoa are governed by a relationship that may hold the key to explaining how disorders of the ciliogenesis process, defined by miR-34/449, ultimately lead to infertility. Spermatozoa are equipped with a single-motile cilia which is called flagellum. Considering the role of miR-34/449 family members during ciliogenesis, and taking into account that spermatozoa represent a special type of ciliated cells, it is of value to analyze the role of miR-34/449 family members on the physiological events entailed during spermatogenesis. The role of miR-34/449 family members on the physiological events entailed during spermatogenesis is summarized in **Figure 1**.

The miR-34/449 family members are expressed in reproductive organs (60–62). Transcriptome analysis in primates and rodents revealed that the miR-34/449 family members are highly expressed in the mature testis, indicating their possible role during sperm maturation (36, 63, 64). In adult male mice, the expression of both miR-34b/c and miR-449a/b/c clusters is progressively increased in the postnatal testis. In contrast, the miR-34a expression seems to be stable during spermatogenesis. This finding could be attributed to the fact that, in the mouse testis, miR-34a is predominantly expressed in spermatogonia rather than in spermatocytes or spermatids (19, 62). In addition, both miR-34b/c and miR-449a/b/c clusters present similar expression patterns in male germ cells. They are considered to be meiotic specific microRNAs as their expression is relatively higher at the initiation of meiotic divisions during spermatogenesis (19, 65, 66).

Considering their expression partners, researchers attempted to investigate the molecular mechanism *via* which the miR-34/449 family members contribute to generation of the mature spermatozoa. Using bioinformatic analysis, several gene-targets were identified, including CDKs, Notch1, Bcl-2 and Casp3. These molecules are known to play essential roles during spermatogenesis (36, 64, 67–69). For example, in the mouse testis Notch1 downregulation is required for optimal spermatozoa maturation. In contrast, the activation of the Notch signaling pathway in mutant mice resulted in impaired spermatogenesis. These mutant mice experienced an age-related sperm count reduction and were also presenting with sperm morphological defects. This defective spermatogenesis, noted in the mutant mice overexpressing Notch, was principally attributed to the inability of the germ cells to properly differentiate to mature spermatozoa. Moreover, Notch overexpression increased apoptosis levels in spermatogonia of the mutant mice (70). To elaborate on that, miR-34b/c and miR-449a/b/c clusters’ members have been characterized as principal post-transcriptional controllers for the transition of germ cells from the pro-meiotic to the meiotic stage (66). These microRNAs target several transcripts of cell-cycle regulatory proteins, leading germ cells to exit the cell cycle, in order for spermatogenesis to be initiated. Interestingly, experiments performing luciferase report assays demonstrated that, in germ cells, the miR-34b/c and miR-449a/b/c clusters’ members are sharing common targets. Some of them include different types of cyclins and CDKs as well as elements of the RB/E2F pathway. The significant role of miR-34b/c and miR-449a/b/c clusters’ members during spermatogenesis is highlighted in experiments performed on animal models presenting with chemotherapy-induced azoospermia. A significant reduction in the expression levels of miR-34b/c and miR-449a/b/c was observed in rats following treatment with busulfan, leading to azoospermia. Following chemotherapy some of the affected rats were subjected to mesenchymal stem cell (MSC) transplantation in the testis. Notably, MSC transplantation was able not only to restore fertility but also to upregulate the previously altered expression of the miR-34b/c and miR-449a/b/c clusters’ members. Following regulation of these microRNAs expression, expression patterns of several other genes, including Cdk1, E2F1, c-Myc and PLCXD3 was normalized (71). This data suggests that miR-34b/c and miR-449a/b/c regulate sperm maturation by controlling expression levels of several proteins involved in spermatogenesis. For instance, and as previously mentioned, the RB/E2F pathway is controlled by the miR-34b/c and miR-449a/b/c clusters’ members during cell cycle control. During spermatogenesis, the E2F1 protein, being a transcription factor, is essential for the maintenance of spermatogonia in a proliferative status, promoting mitotic divisions of these germ cells. However, in later stages of spermatogenesis, the transition of germ cells from the pro-meiotic to the meiotic stage requires silence of the RB/E2F pathway. Upon miR-34b/c and miR-449a/b/c dysregulation, the E2F1 activity during the later stages of spermatogenesis results in Sertoli cell dysfunction, as well as in a progressive reduction of the number of mature spermatocytes and spermatids (72, 73). Moreover, studies indicate that miR-34b stands as a significant controller of spermatogenesis, as it targets the mRNA of the cyclin dependent kinase 6 (Cdk6). It is well established that Cdk6 protein plays crucial roles during spermatogenesis, enabling germ cells to exit the cell cycle in order to differentiate to mature spermatozoa. Indeed, overexpression of the miR-34b in cultured cells significantly downregulated expression levels of the Cdk6 protein (74). Similar studies indicate that also miR-449 mimics could lead to proliferation inhibition (19). MicroRNA-34/449 family members are essential molecules promoting normal spermatogenesis *via* the differential regulation of expression levels of several spermatogenesis-related proteins depending on spermatogenesis stages.

The crucial contribution of miR-34/449 family members during spermatogenesis is demonstrated in several studies investigating spermatogenesis outcomes upon the absence of these specific microRNAs. Knockdown male mice for either miR-34b/c or miR-449a/b/c clusters, presented with no significant spermatogenic defects and were phenotypically normal. In both cases, the knockdown male mice had normal fertility and normal sperm parameters and were also presenting with normal testicular morphology. The normal phenotype of the single knockdown mice could be attributed to the fact that miR-34b/c or miR-449a/b/c clusters are sharing common targets and roles. Interestingly, upon miR-449a/b/c cluster’s genetic ablation, an upregulation of miR-34b/c expression was observed, confirming the synergetic action of these microRNAs (19, 60). However, simultaneous inactivation of both clusters resulted to male infertility while mice presented with a phenotype resembling OAT. Sperm analysis revealed impaired spermatogenesis and both the number, and the motility of the spermatozoa were detrimentally affected. Spermatozoa found in the epididymis were headless to an extent of 80%. Image analysis employing transmission electron microscopy revealed major defects in the formation of the flagellum architecture. The defects included a disorganization of the mitochondrial sheath and the lack of the typical “9+2” microtubular organization. However, and despite the major structural defects of the mature spermatozoa, mutant round spermatids showed normal fertility potential and the lack of the miR-34b/c and miR-449a/b/c clusters did not affect development of the generated embryos (25, 60). In light of the above, miR-34/449 family members do not only exert significant actions for the regulation of spermatogenesis, but are also essential for proper formation as well as for proper function of spermatozoa per se.

Current data also demonstrate that miR-34/449 family members represent significant regulators of the molecular mechanisms involved in maintaining testicular homeostasis. During spermatogenesis, miR-34/449 family members induce apoptosis, eliminating defective cells, including abnormal germ cells and immature spermatozoa. *In vitro* experiments employing GC-2 lines highlight that miR-34c possibly regulates apoptosis through repression of the ATF1 mRNA (75). In addition, the miR-34a seems to play crucial roles for oxidative stress mediated apoptosis, inhibiting the Sirt1 protein in the testis. The induction of oxidative stress in the mouse testis results in an upregulation of miR-34a and downregulation of SIRT1. Upon oxidative stress, the testis displays higher expression of apoptotic markers and higher apoptotic index in male germ cells, indicating that oxidative stress is a major cause of male infertility (76, 77). The members of the miR-34/449 family seems to exert significant anti-oxidant and anti-apoptotic properties and are vital for the testicular homeostatic mechanisms. Upregulation of their expression observed upon oxidative stress indicates that members of the miR-34/449 family ensure-via their unique actions-maintenance of spermatogenesis when the microenvironment of the testis is jeopardized. Thus, alterations with regard to miR-34/449 expression have been correlated with defective anti-oxidant capacity, ultimately leading to male infertility (78).

## Male Infertility in Humans and miR-34/449 Associations

Similarly to animal models, several studies suggest that in humans the dysregulated expression of miR-34/449 family members is strongly associated with failed spermatogenesis, with impaired testicular functionality and subsequently with male infertility (79–82). Considering the expression motifs of the miR-34/449 family members in the male reproductive system, data indicate that these microRNAs are expressed in both spermatozoa and testicular tissues but not in the accessory glands, highlighting the possible implication of miR-34/449 family in male infertility originating from testicular dysfunction (83).

More specifically, studies investigating microRNAs’ expression patterns in patients with male infertility, revealed that members of the miR-34b/c were significantly downregulated in both spermatozoa and in seminal plasma of men presenting with oligozoospermia and azoospermia (79–82). Between the two members of the miR-34b/c cluster, miR-34b was positively correlated with spermatozoa vitality and concentration (81, 82). Furthermore, it is demonstrated that miR-34b levels are significantly reduced in spermatozoa as well as in testicular samples of men presenting with different types of NOA, in comparison to men with normal spermatogenesis. Analysis employing receiver operating characteristic curve (AUC) indicated that miR-34b could successfully separate different types of NOA and control fertile men, as it presented with a high AUC value reaching 0.944 (95% CI 0.9131–0.9713) (80). In concordance to the aforementioned studies, Salas Huetos et al. indicated that miR-34b levels were significantly reduced in semen samples of oligozoospermic and asthenozoospermic men. This is a significant observation considering that semen could be used as a material for non-invasive differential diagnosis of male infertility compared with tissue analysis, which requires invasive testicular biopsies. In the same study, bioinformatic analysis revealed altered expression patterns of several genes involved in spermatogenesis, chromatin modification and cell cycle regulation, highlighting that several of these genes are targets of the miR-34b/c. These data are of paramount importance for better understanding the connection between the miR-34/449 alterations with male infertility. As anticipated, considering the shared mechanisms of action between miR-34b/c and miR-449a/b/c clusters, the miR-449a/b/c cluster also displays similar expression patterns as the miR-34b/c cluster’s members. These entail decreased levels in semen samples of infertile patients presenting with abnormal semen parameters (84). When critically analyzing data, it appears that there is a great body of evidence suggesting that the altered expression of the miR-34/449 family members is strongly associated with several aspects of male infertility ranging from oligozoospermia to NOA. Most importantly, miR-34/449 levels in spermatozoa, in testicular tissues as well as in seminal plasma seem to be directly associated with severity of male infertility. Thus, miR-34/449 family members could serve as potential sensitive biomarkers for an accurate individualized differential diagnosis, as well as for assessment of male infertility severity.

It has been noted that upon miR-34/449 dysregulation both testicular functionality and spermatogenesis are severely compromised, commonly leading to idiopathic male infertility. However, the exact mechanisms involved in miR-34/449 dysregulation in infertile men presenting with impaired spermatogenesis as well as with diminished testicular functionality are poorly understood. Current evidence suggests that epigenetic alterations, including abnormal methylation of the miR-34/449 loci, probably stands as one of the main pathological mechanisms inducing abnormal miR-34/449 expression patterns. Indeed, two recently published studies reported a higher methylation status in the promoters of miR-34b/c and miR-449a/b/c clusters in men presenting with idiopathic male infertility and abnormal semen parameters (84, 85). In both studies, infertile men presented with a higher percentage of methylation in the miR-34/449 promoters and subsequently with a significant reduction of the expression levels of miR-34/449 compared with fertile men featuring normal semen parameters. Interestingly, these alterations in miR-34/449 methylation were documented as directly associated with the severity of semen abnormalities, with OAT patients presenting with the highest methylation percentage, as well as the lowest miR-34/449 levels (84, 85). Moreover, defective genomic imprinting of miR-34/449 loci was also observed in patients presenting with male infertility (86–88). The underlying causes of altered imprinting as well as of defective methylation of the miR-34/449 loci are yet poorly understood. In the study of Najafipour and colleagues it was suggested that smoking can increase the methylation status of the miR-449 cluster’s promoter (84). Other researchers have similarly associated smoking with aberrant methylation patterns in several genes as well as with abnormal semen parameters, indicating the detrimental epigenetic effect of smoking on male fertility (89–91). Evidence suggests that several other lifestyle parameters, physical activity levels, stress as well as aging are described as factors that could affect DNA methylome in spermatozoa, leading to abnormal methylation patterns in specific genome loci, including the miR-34/449 loci, eventually leading to structural and functional abnormalities of spermatozoa (86). For example, there are studies reporting an age-related reduction of miR-34b levels, indicating that aging could negatively affect male reproductive potential (Salas-Huetos et al., 2019). It has also been voiced that early life stress is associated with reduction of miR-34/449 levels in semen samples. Men with high scores of early life stress showed a reduced expression of miR-34/449 in semen, coupled by poorer semen parameters in comparison to individuals experiencing low early life stress (92). These data indicate that stress could impair miR-34/449 expression, reducing male reproductive capacity, however the underlying mechanisms remain unknown (93). Except from the epigenetic alterations, several genetic and chromosomal abnormalities have been also associated with impaired miR-34/449 expression. For instance, interesting results emerge from a recently published study, where the expression profile of several microRNAs in the testes of patients with Klinefelter (46, XXY) syndrome were investigated (94). It is well-known that individuals with Klinefelter syndrome are commonly presenting with NOA. Results of this study revealed a significant reduction of miR-34b/c in testicular samples of Klinefelter patients compared with individuals diagnosed with obstructive azoospermia. Notably, miR-449 cluster’s members were undetectable in the testicular samples obtained from Klinefelter patients (94). These interesting findings suggest, that despite the fact that miR-34/449 are encoded from transcriptional units located in autosomal chromosomes, their expression is possibly regulated by factors encoded from loci on the sex chromosomes. Alterations in sex chromosomes could detrimentally affect miR-34/449 expression patterns, highlighting the need for further research to better understand the complex network regulating the miR-34/449 family. Apparently, more data are required in order to unveil the pathophysiological mechanisms leading to miR-34/449 alterations, which in turn cause male factor infertility. Several inherited or/and acquired genetic and epigenetic conditions, such as genome imprinting and DNA methylation, have been so far correlated with impaired miR-34/449 expression patterns. It is of high clinical significance to note that life-style parameters, environmental stressors as well as aging, are at the top of the list of the cascade of events leading to miR-34/449 dysregulation and to male infertility.

Considering the significant role of miR-34/449 in idiopathic male infertility, it is of added value to further study the possible implication of these microRNAs on pathologies that represent known causes of male infertility, such as varicocele. It is well-established that varicocele is associated with increased testicular temperature, leading to germ cell damage and finally to temperature-depending spermatogenic failure (95). Several molecular mechanisms are involved in heat-induced germ cell damage, including apoptosis, oxidative stress, DNA damage and autophagy (95). Indeed, a significant reduction of miR-34c levels has been reported in patients presenting with varicocele and impaired semen parameters in comparison to fertile men with varicocele and normal testicular functionality. In the same study, miR-34c levels were positively correlated with semen parameters, namely spermatozoa concentration, motility and morphology. In contrast, miR-34c levels were negatively correlated with markers indicating oxidative stress and apoptosis (96). Lower levels of miR-34a were also reported in varicocele patients, according to the results provided by a recently published study, which similarly suggests that miR-34/449 family dysregulation constitutes part of varicocele’s pathophysiology. More specifically, varicocele patients presented with reduced expression of miR-34a as well as with increased levels of oxidative stress markers in their semen samples, compared with healthy fertile controls (93). In order to connect the molecular events entailed in varicocele pathophysiology with the miR-34/449 family, we should consider that these microRNAs exert anti-apoptotic properties (97). Moreover, there are reports suggesting that miR-34/449 family members also serve as oxidative stress-responsive elements (98). In light of that it becomes evident that miR-34/449 dysregulation could be associated with increased germ cell apoptosis as well as with increased oxidative stress in the testis of varicocele patients. Notably, data also demonstrate that the levels of miR-34/449 family members are strongly associated with the severity of impairment observed in semen parameters of varicocele patients, highlighting their possible role as biomarkers for properly categorizing these patients. In addition, miR-34/449 members may stand as a possible non-invasive and reliable tool. This in the future could be employed to assist andrologists in successfully assessing the reproductive potential of varicocele patients subjected to surgical treatment, in a personalized, patient-friendly and precise manner. However, future studies are needed to verify the value of this hypothesis.

As discussed in previous sections of this manuscript, the miR-34/449 family is implicated in several aspects of male infertility. At this point, authors focused on presenting the current evidence regarding the role of miR-34/449 family members on the pathophysiological mechanisms involved in azoospermia, which represents by far the most severe expression of male infertility. Azoospermia is characterized by the absence of spermatozoa in the ejaculate and is a well-studied condition (99). Data indicate that azoospermia is a multifactorial condition caused by a wide variety of congenital, acquired, and idiopathic factors, including genetic, anatomical, endocrine, as well as environmental factors (100–102). However, for a large proportion of azoospermic patients the exact causes of azoospermia cannot be identified, a condition called idiopathic azoospermia (103). Management of these patients is highly challenging, especially with regards to NOA, where histological examination of testicular tissues can reveal different types of spermatogenic failure, ranging from a mild hypo-spermatogenesis to complete loss of spermatozoa, a syndrome called Sertoli Cell Only (SCO) syndrome (104, 105). Considering the pathophysiology of azoospermia, data originating from several studies indicate the possible role of several microRNAs on the molecular basis of testicular impairment observed in this condition (82, 106–108). Numerous studies indicate abnormal expression patterns of several microRNAs in patients with azoospermia (107, 109–111). Notably, miR-34/449 family members seem to represent the most affected class of microRNAs. In 2009 Lian and colleagues were the first to examine the microRNAs’ profile in NOA patients and discovered multiple alterations in the expression patterns of several microRNAs in the testicular tissues obtained from these patients. Results also highlighted that both miR-34b and miR-449a were significantly under-expressed in NOA patients (109). Following this evidence, a series of similar studies was conducted, where more data on the distinct microRNA expression patterns of azoospermic patients was sourced. In studies, where microarray analysis was employed on testicular biopsies of patients presenting with different subtypes of NOA diagnosis, including SCO syndrome, mixed atrophy and germ cell arrest, it was revealed that, in all of the aforementioned groups, both miR-34b/c and miR-449a/b/c clusters were the most downregulated class out of all the examined microRNAs (112). Interestingly, miR-34b/c and miR-449a/b/c clusters seem to exert different expression profiles according to the type of NOA. This is indicated by the study of Munoz et al., where the reduction observed in miR-34/449 levels was higher in patients with SCO syndrome compared to patients that presented with maturation arrest. These interesting findings suggest that miR-34/449 family members exert significant regulating actions, which are a prerequisite for germ cell differentiation, especially following the beginning of meiosis during testicular development as well as in adult spermatogenesis (19, 94). Other studies also highlight these observations (82). At this point it is interesting to note that despite the differences, NOA subgroups are sharing common downregulated microRNAs, which underlies that NOA conditions may possibly arise from identical molecular alterations (107). To conclude, evidence suggest that miR-34/449 family members play crucial roles on azoospermia pathogenesis. Alterations of miR-34/449 expression patterns are associated with impaired spermatogenesis, especially following the stage, where meiotic divisions are initiated. Expression patterns of the miR-34/449 family merit further investigation to be showcased as an effective tool for better categorizing NOA patients.

Regarding NOA caused by spermatogenic failure, the gold standard management is sperm retrieval employing micro-testicular sperm extraction (TESE). However, TESE fails to ascertain a high percentage of success resulting in emotional distress for couples (113). It is imperative to develop reliable and non-invasive markers that could reflect not only testicular histopathology, but that could accurately predict success of a sperm retrieval. The miR-34 family members could be a promising predictive molecule for the clinician alone or in combination with other miRNAs. It is interesting to note that when miR-34b and miR-10b were combined as predictive biomarkers, the ROC curve analysis indicated a high predictive diagnostic accuracy in distinguishing NOA from fertile individuals (114). Additionally, Fang et al. investigated and compared the microRNA profiles of NOA patients with successful and unsuccessful sperm retrieval. They found that the two groups had 180 differentially expressed microRNAs, with miR-34 and miR-449 clusters being the most downregulated in testicular biopsies and seminal plasma of the unsuccessful sperm retrieval group (115). These significant data demonstrate that these microRNAs could not only be used for the accurate diagnosis of NOA, but also for prognosis of TESE effectiveness.

## Discussion

**Figure 2:**
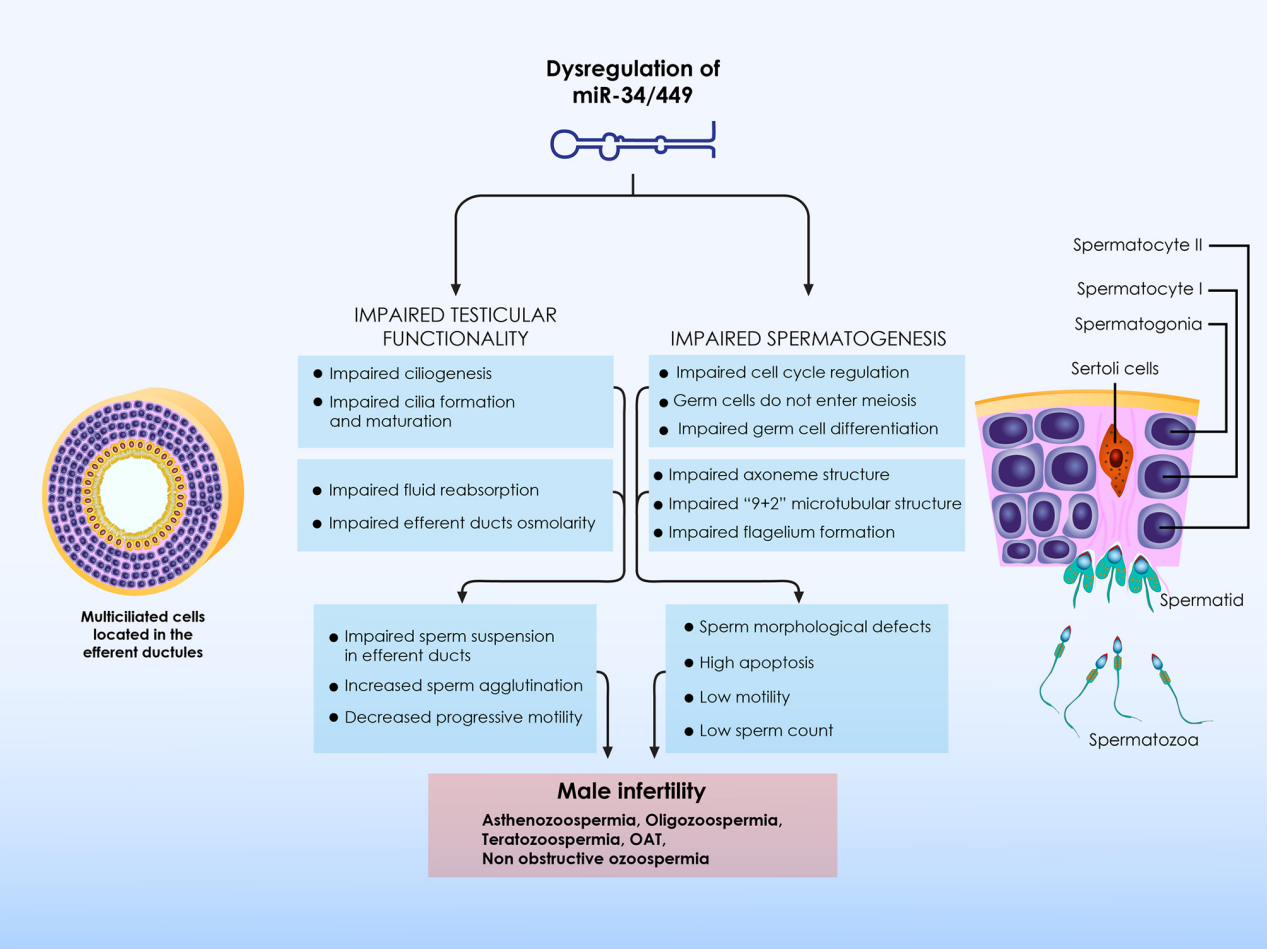
Summary of the role of miR-34/449 on male infertility. Alterations in the expression patterns as well as in functionality of miR-34/449 family members detrimentally affect male reproductive potential, impairing both testicular functionality as well as spermatogenesis per se. On the one hand, impaired miR-34/449 expression patterns result to defective ciliogenesis in the efferent ductules epithelium, leading to impaired reabsorption of the seminiferous tubular fluids. Failed reabsorption of the fluids in turns leads to epididymal spermatozoa agglutination and aggregation. In these conditions the number of spermatozoa reaching to caput epididymis is significantly reduced and as a result the number of mature motile spermatozoa is significantly decreased causing OAT or even azoospermia. Impaired ciliogenesis also leads to fluid accumulation into the rete testis, which in turns lead to high hydrostatic pressure into the testis. Hydrostatic pressure progressively leads to spermatogenic failure, finally resulting to male infertility. On the other hand, alterations regarding miR-34/449 expression patterns also directly affect spermatogenesis. Upon miR-34/449 dysregulation, the expression patterns of several spermatogenesis-related proteins are disrupted, preventing germ cells to properly enter meiosis and thus to differentiate to mature spermatozoa. Moreover, miR-34/449 dysregulation also directly impairs spermatozoa formation and maturation, mainly considering flagellum construction and function. Thus, alterations with regard to miR-34/449 expression patterns are directly associated with impaired semen parameters, including abnormal spermatozoa concentration, morphology and motility. *OAT, oligoasthenoteratozoospermia*.

This review is all-inclusively presenting current data on the implication of the miR-34/449 family members in male infertility. Evidence provided is summarized in **Figure 2** and highlights that alterations in expression patterns as well as in functionality of the miR-34/449 family members detrimentally affect male reproductive potential, impairing both testicular functionality as well as spermatogenesis *per se*.

In the present review authors highlight that altered miR-34/449 expression patterns can cause defective ciliogenesis in the efferent ductules epithelium, leading to progressive spermatogenic failure, which could range from OAT to azoospermia. In this case, spermatogenic failure is attributed to testicular dysfunction, resulting from impaired reabsorption of the seminiferous tubular fluids, which in turn leads to high hydrostatic testicular pressure. These cases, where multicillia cell dysfunction and efferent ductules obstruction occur, are characterized by a unique phenotype, where abnormal semen parameters and/or azoospermia co-exist with sperm aggregation and agglutination, as well as with luminal enlargement. The clinical significance of these findings is of paramount importance, considering that these abnormalities resemble the condition of NOA as well as of the SCO syndrome. Considering the small size of the efferent ductules as well as the topographic anatomy of this organ, we can assume that efferent ductules’ obstructions could-in some cases-be overseen, bypassed and misdiagnosed. Indeed, there are reports indicating that patients presenting with immotile cilia syndrome also present with azoospermia (116, 117). This could be attributed to the efferent ductules’ obstruction in these patients, resulting to fluid accumulation and subsequently generation of testicular hydrostatic pressure. Proper diagnosis of these cases is of value since surgical interventions may provide an effective way for relieving hydrostatic pressure, aiming to improve testicular functionality in order to recover sperm production and restore-to some extent-male fertility (27).

This review further highlights data demonstrating that miR-34/449 family members also constitute significant regulators of spermatogenesis. These microRNAs control expression of several regulatory proteins and are essential for proper transition of the germ cells from the pro-meiotic to meiotic stages. Most importantly, these microRNAs play crucial roles during the final sperm maturation, and upon their dysregulation both spermatozoa formation, as well as functionality are jeopardized. Their added value becomes evident considering that their expression is significantly correlated with semen parameters, including spermatozoa concentration, morphology and motility. In the future, these microRNAs may be employed as specific biomarkers assisting clinicians to in diagnose, and design an efficient management protocol. This may be of value especially in cases of idiopathic male infertility. We herein also demonstrate that miR-34/449 could not only be used to distinguish fertile from infertile men, but also to perform a non-invasive accurate diagnosis defining male infertility etiology, especially regarding NOA patients. Today, male factor infertility diagnosis and evaluation principally includes medical history assessment, physical examination, hormonal assessment and semen analysis. Under specific circumstances, further assessment may be required, including genetic testing, imaging analysis such as scrotal ultrasonography and specific semen testing such as sperm DNA fragmentation testing (3). However, and despite the great advances observed in the field considering development of a variety of diagnostic tools, their interpretation is often imprecise, subjective, vague and inaccurate, highlighting the need for more precise diagnostic tools (118). Data suggest that miR-34/449 members may serve as such biomarkers.

Data summarized herein indicate that miR-34/449 family members are implicated in male reproductive system physiology and pathology. The question is “which physiological mechanisms and factors regulate their expression patterns?” In a recently published review, Ioannis Loukas and colleagues summarize the current evidence with regard to the molecular mechanisms and factors regulating miR-34/449 expression levels during ciliogenesis (119). Authors highlight that both tumour suppressor protein P53 and E2 promoter binding factor 1 (E2F1) stimulate miR-34/449 expression, however their role during multiciliogenesis remains unclear (119–121). The transcriptional domain-containing active p73 (TAp73) has been recently introduced as a master regulator of multiciliogenesis process in several tissues and organs (119, 122, 123). This factor directly binds to miR-34b/c loci and transactivates the expression of this specific microRNA cluster. However, it seems that p73 is differentially expressed among the different multiciliated tissues, as its expression is not a prerequisite for multicillia cell differentiation in either the respiratory or the reproductive track epithelia (124). These data support the hypothesis that alternative pathways have been developed in diverse tissues to support multicillia cell differentiation (119). Geminin superfamily and more specifically GemC1 and McIdas have been recently introduced as key-regulators of multiciliogenesis in the airway epithelium as well as in the brain (37, 44, 125, 126). Interestingly, GemC1 constitutes a predicted miR-34/449 target, however their complex interactions during ciliogenesis remain unclear (119). Considering the aforementioned, the possible role of Geminin family during multicillia cell differentiation in other tissues and organs, including the testis and the fallopian tubes, merits further investigation. Focusing on testicular functionality, limited data exist with regard to the upstream elements controlling miR-34/449 expression patterns. It seems that two transcription factors, namely cAMP-responsive element modulator τ (CREM) and SRY-box transcription factor 5 (SOX5), serve as upstream regulators and are able to transactivate miR-449 cluster in the mouse testes (19). Interestingly, both of these factors also regulate male germ cell gene expression patterns, and their dysregulation is strongly associated with severe male factor infertility, such as NOA (127). In summary, investigation of the upstream pathways controlling miR-34/449 expression patterns in the testis may open a new line of investigation, unveiling potential biomarkers as well as novel therapeutic targets, towards better understanding and efficiently managing male infertility.

Considering the clinical perspective in addressing male infertility, significant information could be retrieved by analyzing miR-34/449 levels in both testicular tissues and semen, namely predicting a successful sperm retrieval following biopsy. The prognostic value of these microRNAs could ascertain improvement of *in vitro* fertilization/intracytoplasmic sperm injection (IVF/ICSI) outcomes, reducing the cost as well as well as psychological discomfort associated with male infertility (128). The significance of these findings is highlighted, considering that infertility is not strictly a medical issue but rather extends to social, psychological, and even bioethical aspects. An infertility diagnosis impacts both partners on various levels, entailing psychological distress and financial strain as treatment may be costly. National Health systems-where involved-may equally be financially burdened (129, 130). The need to provide solutions through research in addressing infertility, from diagnosis to treatment has been thoroughly documented (129). Thus, identifying biomarkers that allow proper patient categorization is a powerful tool in the era of personalized and precision medicine.

Taking a closer look into the advanced insemination techniques employed today to address male infertility, as part of our clinical routine tools we identify ICSI and intracytoplasmic morphologically selected sperm injection (IMSI), and development of accurate surgical interventions for sperm collection direct from the testis, such as testicular sperm aspiration (TESA) and TESE. These options have led clinicians and clinical embryologists to successfully overcome several types of male infertility, including the most severe namely azoospermia. Coupled by high efficiency, these techniques were quickly adopted by clinicians and fertility centers all over the word (131). Despite several couples achieving their reproductive goals employing these state-of-the-art techniques, it should be highlighted that these techniques and methods have been designed to merely bypass the barrier of male factor infertility, albeit the exact infertility aetiology remains untreated. Several reports highlight that perhaps availability and-to some extent-overuse of these advanced techniques lessens the urgency for investigating in depth treatment options, and improving male fertility status at its core (132). Even though advanced assisted reproductive technology (ART) techniques can circumvent male infertility and provide solutions for fertilizing an oocyte, nonetheless, the solution to properly address and treat male infertility still eludes us. It appears that research is more clinically driven and focused on addressing the “symptom”, rather than investigating means to understand and treat the actual condition. Providing clinically driven solutions fails to effectively address male infertility in depth. This should be taken into consideration as there is a great body of evidence suggesting that sperm quality as well as paternal health at the time of conception could significantly affect outcomes (133, 134). These outcomes range from the IVF treatment’s efficiency, to embryo development, pregnancy, and finally to the offspring’s’ health, and even the children’s future reproductive potential (133, 134). Data also indicate that harmful epigenetic modifications could be inherited from damaged spermatozoa to the next generation (135). To add to that, several studies suggest that embryos resulting from spermatozoa of impaired quality are characterized by reduced developmental and implantation potential, increasing the incidence of pregnancy failure (136, 137).

Taking into account this critical analysis, it is of paramount importance to focus not only on developing advanced technologies aiming to override the barriers of male infertility, but most importantly to focus on performing research to understand male infertility pathophysiology in depth. Better understanding the pathological basis of male infertility, in a personalized and precise manner, and following on the concept of individualization and precision could significantly assist the reproductive scientist. Orienting research to develop higher performance diagnostic and evaluating tools, will enable design of accurate, targeted and efficient therapies, in order to personalize patients’ management and finally to ensure the health of future generations. Only by aspiring to understand and treat the condition of male infertility itself, and not merely bypass it, we may advance towards ascertaining health of the next generations. It is this realization that fuels and justifies research on identifying areas focusing on novel molecular aspects of male infertility. This review uniquely presents the case of miR-34/449 family members, all-inclusively describing their involvement while showcasing their value in investigating and addressing male infertility.

## Author Contributions

KP, SG, KS, and MS conceived and designed the project. SG, PT, and IL performed the literature review. KP, SG, PT, IL, EM, AP, NN, TV, and GK contributed to drafting and editing the manuscript. KP, SG, GK, AA, KS, and MS revised the manuscript. GK, AA, KS, and MS contributed to the critical discussion and provided intellectual content. KP, KS, and MS supervised the study. All authors contributed to the article and approved the submitted version.

## Conflict of Interest

The authors declare that the research was conducted in the absence of any commercial or financial relationships that could be construed as a potential conflict of interest.
